# Notch1/TAZ axis promotes aerobic glycolysis and immune escape in lung cancer

**DOI:** 10.1038/s41419-021-04124-6

**Published:** 2021-09-04

**Authors:** Mian Xie, Xin-ge Fu, Ke Jiang

**Affiliations:** 1 Department of Medical Oncology, Guangdong Provincial People’s Hospital and Guangdong Academy of Medical Sciences, Guangzhou, China; 2 Department of Pathology, The First Affiliated Hospital of Guangzhou Medical University, Guangzhou, China; 3 Department of Internal Medicine, The First Affiliated Hospital of Guangzhou Medical University, Guangzhou, China

**Keywords:** Cancer, Cancer

## Abstract

Oncogenic signaling pathway reprograms cancer cell metabolism to promote aerobic glycolysis in favor of tumor growth. The ability of cancer cells to evade immunosurveillance and the role of metabolic regulators in T-cell functions suggest that oncogene-induced metabolic reprogramming may be linked to immune escape. Notch1 signaling, dysregulated in lung cancer, is correlated with increased glycolysis. Herein, we demonstrate in lung cancer that Notch1 promotes glycolytic gene expression through functional interaction with histone acetyltransferases p300 and pCAF. Notch1 signaling forms a positive feedback loop with TAZ. Notch1 transcriptional activity was increased in the presence of TAZ and the activation was TEAD1 independent. Notably, aerobic glycolysis was critical for Notch1/TAZ axis modulation of lung cancer growth in vitro and in vivo. Increased level of extracellular lactate via Notch1/TAZ axis inhibited cytotoxic T-cell activity, leading to the invasive characteristic of lung cancer cells. Interaction between Notch1 and TAZ promoted aerobic glycolysis and immune escape in lung cancer. Our findings provide potential therapeutic targets against Notch1 and TAZ and would be important for clinical translation in lung cancer.

## Introduction

Notch signaling exerts oncogenic and tumor-suppressive effects during tumorigenesis. In human, there are four Notch receptors (Notch 1–4) that, upon interaction with Notch ligand DLL1, DLL3, DLL4, Jagged1, and Jagged2, are proteolytically cleaved to release Notch intracellular domain (Notch ICD), which translocates into the nucleus to regulate the expressions of target genes, including Hes1 [1]. Our previous study shows that Notch1 overexpression is associated with drug resistance in non-small cell lung cancer (NSCLC) [2]. In lung cancer, Notch1 co-expression is distinctly enriched for pathways associated with angiogenesis and vascular development, immune system, and Rho GTPase activity [3]. Notch1 is involved in the innate immune system and Notch1 knockdown in lung cancer enhances innate immune recruitment [4].

Recent study shows that tumor metabolic dysregulation sensitizes cancer cells to antitumor immune cells implying that Notch-associated metabolic phenotype and immune escape harmonize during cancer progression [5]. Lung cancers demonstrate enhanced aerobic glycolysis and microenvironmental acidity [6]. Lung cancer cells produce large amounts of lactate regardless of oxygen levels. Tumor acidity is a key regulator of cancer immunity that orchestrates immunosuppression [7].

Our studies find that Hippo effector transcriptional coactivator with PDZ-binding motif (TAZ) is overexpressed in lung cancer and associated with poor survival and defines a clinically distinct subgroup of patients with NSCLC [8, 9]. TAZ promotes cell growth through a transcriptional program mediated by the transcriptional enhanced associate domain (TEAD) transcription factors [10]. Transcriptional coactivator with PDZ-binding motif (TAZ) is a YAP homolog that is also tightly regulated by the Hippo pathway. YAP/TAZ is also involved in metabolism regulation such as to promote glycolysis [11]. The key enzyme of glycolysis, phosphofructokinase 1 (PFK1), and YAP/TAZ form a complex in the nucleus and promote the malignant biological behavior of cancer cells [12].

The crosstalk between YAP/TAZ and Notch signaling influences self-renewal, stem cell differentiation, cell fate decisions, and tumor progression [13]. However, the molecular link between Notch and TAZ involved in the immune escape via glycolysis in lung cancer adenocarcinoma remains obscure. Here we report a positive feedback loop of Notch1 and TAZ in lung cancer cells that increases lactate production, which promotes immune escape.

## Materials and methods

### Cell culture, transfection, and drug treatment

A549 and PC9 cell line were obtained from the American Type Culture Collection (ATCC, Manassas, VA). Cell lines were authenticated using the sequence-tagged site (STS) fingerprinting and cultured in RPMI 1640 supplemented with 10% fetal bovine serum (Sigma, St. Louis, MO). shRNAs targeting Notch1, pCAF, p300, TAZ, or TEAD1, and their respective negative control RNAs were purchased from Santa Cruz Biotechnology (CA, USA). Transfection was performed using Lipofectamine 2000 (Invitrogen) according to the manufacturer’s instructions. Stable transfectants were selected by 800 μg/mL of G418 (Sigma, US) for 1 week and maintained with 100 μg/mL of G418. Cells were treated with 2.5 mM 2-DG or 0.1 mM oligomycin at indicated times. Human primary CD8+ cells were isolated from peripheral blood mononuclear cells using the CD8+ cell isolation kit (Miltenyi Biotec Co., US). 2 × 10^5^ CD8+ cells were added into A549 cells at 7:1 radio in the presence of 10 mmol/L lactate for 96 h at 37 °C.

Tumor-infiltrating leukocytes were isolated from A549 xenograft tumors. Briefly, mechanically dissociated tissues underwent enzymatic digestion with hyaluronidase and collagenase type IV. Single-cell suspensions were filtered through a 100 μm cell strainer (BD Co., CA), washed, and mononuclear cells were prepared by Percoll density gradient centrifugation.

### Plasmid constructs

The Notch1 intracellular domain (Notch1 ICD) was generated by subcloning the activated Notch1 intracellular domain into the pcDNA3-HA2 vector as previously described [1]. Human TAZ and Mst1 cDNAs were cloned into the p3xFlag-CMV-14 expression vector (Sigma) to construct p3xFlag-TAZ and p3xFlag-Mst1 vectors, respectively. All the constructs were confirmed by DNA sequencing. For reporters, pRL-TK-Notch1, pRL-TK-Hes1, and pRL-TK-Jagged1, human wild-type Notch1, Hes1, or Jagged1 were cloned downstream of the Renilla luciferase coding sequences in pRL-TK (Promega, CA, US).

### Luciferase reporter assay

Luciferase reporter assays were performed according to the manufacturer’s instructions (Promega Inc., CA, US). The Notch1 promoter construct (−2001/−1) was generated from human genomic DNA corresponding to the sequence from −2001 to −1 (relative to the transcriptional start site) and cloned to the pRL-TK-Basic vector (Promega, Madison, WI, USA). The Hes1 promoter construct (−2000/+100) was generated from human genomic DNA corresponding to the sequence from −2000/+100 (relative to the transcriptional start site) and cloned to the pRL-TK-Basic vector (Promega, Madison, WI, USA). The Notch1 or Hes1 promoter plasmid or its negative control basic plasmid carrying the firefly luciferase reporter were co-transfected with an internal control, pRL-TK Renilla vector (Promega), by using Lipofectamine 2000 (Invitrogen). Briefly, 1 × 10^4^ cells were seeded in 24-well plates. For analysis of Notch1 and Hes1 promoter activity, 3 × 10^6^ cells plated in 24-well plates and were co-transfected with 40 ng of reporter construct, 500 ng of expression vector, and 5 ng of the internal control Renilla construct (Promega). Twenty-four hours after transfection, the cells were harvested in 1× lysis buffer and subjected to a single freeze-thaw cycle to ensure complete lysis. Cell lysates were transferred to the microcentrifuge tubes, vortexed for 3 min and then centrifuged at 1.2 × 10^4^ rpm for 5 min at 4 °C. Ten microliters of supernatants were mixed with 10 ml of the Luciferase Assay Reagent per tube and measured for the luciferase activity using the luminometer. Renilla activity was used to normalize luciferase reporter activity.

### Cell proliferation assay

2 × 10^5^ A549 and PC9 cells were transfected with 1 µg of Notch1 alone or co-transfected with 1 µg of Notch1 and TAZ. Cell proliferation was determined by the CCK-8 Kit (Dojindo Laboratories) according to the manufacturer’s instructions. Briefly, 10 µL of CCK-8 solution were added to cultured cells in each well, followed by incubation at 37 °C for 1 h. The OD values were measured at 450 nm using a microplate reader.

### Xenograft assay

Seven-week-old BALB/c athymic nude mice were subcutaneously inoculated in the dorsal left flank with 3 × 10^6^ A549 cells. The experiment was performed as we described previously [1]. In brief, A549 cells were stably transfected with Notch1, TAZ, p300 shRNA, pCAF shRNA, or LDHA shRNA. At 24 h post-transfection, equal numbers of viable cells were injected subcutaneously into nude mice for tumor xenograftment. Tumors were examined every 7 days for a total of 35 days. Tumor growth rates were analyzed by measuring tumor length (L) and width (W) and calculating the volume with the formula LW^2^/2. All animal experiments were conducted in accordance with the approval of the Institutional Animal Care and Use Committee of Guangdong Provincial People’s Hospital.

### MicroPET-CT imaging of mice

MicroPET/CT imaging of mice was performed as we described previously [14]. Xenograft tumor model was established by subcutaneously injected A549 cells transfected with Notch1, or co-transfected with p300 shRNA, pCAF shRNA, or TAZ shRNA. In brief, tumor-bearing mice were placed on a small animal microPET/CT scanner 40 min after injection of 100–200 μCi ^18F^FDG via tail vein. Mice were first subjected to a 15 min microCT scan and then to a 10 min microPET scan. microPET and microCT images were analyzed with Inveon software (Siemens). Regions of interest (ROI) were manually drawn by qualitative assessment covering the entire tumor. Tumor volumes were generated by summing voxels within the tomographic planes. The ROI counts were converted to the %ID/g tumor using filtered back projection.

### Bioluminescence image of in vivo assay

Bioluminescent images were taken with Xenogen IVIS (In Vivo Imaging Solutions) using D-luciferin. Images were normalized using Living Image software (PerkinElmer) with a minimum and maximum radiance of 5 × 10^5^ and 2.5 × 10^6^ photons/s, respectively. The maximum luminescent intensity and total flux in photons per second were calculated and reported for each mouse’s abdominal region in photons/sec. Significance was determined using one-way Anova for luminescence. All time points were compared to the earliest time point of 7 weeks after tumor cell injection. Successful engraftment of subcutaneously xenograft tumors was defined as 5 × 10^5^ photons/s. This value was based on prior studies which demonstrated that failure to achieve more than 10^5^ photons/s bioluminescent emissions correlated with the rejection of implanted tumor [15].

### Western blot and immunohistochemistry assay

Total protein was isolated from cells using Cell Extraction Buffer (Biosource, Camarillo, CA) supplemented with protease and phosphatase inhibitors and precleared using centrifugation, followed by measuring protein concentrations using the BCA Protein Assay kit (Pierce, Rockford, IL). The primary antibodies including TAZ, CTGF, Notch1, Hes1, LDHA, PFKB3, PEPCK, PKM2, PGK1, HK2, GLUT1, GLUT3, ALDOA, p-TAZ (Ser89), and β-actin were purchased from Cell Signaling Technology (CA, US). Jagged1 and TEAD1 were purchased from Santa Cruz Biotechnology (CA, US). Appropriate secondary antibodies conjugated to horseradish peroxidase were used, including anti-mouse or anti-rabbit IgG (GE-Healthcare, CA, US). Proteins were visualized by Amersham enhanced chemiluminescence (GE-Healthcare, CA, US). Immunohistochemistry assay was performed as previously described [7].

### Co-immunoprecipitation (Co-IP)

Co-IP assay was performed as previously described [16]. For transfection-based Co-IP assays, 2 × 10^5^ cells were transfected with the indicated plasmids using Lipofectamine 2000 (Invitrogen, CA, US), lysed in 500 μL of lysis buffer (50 mM Tris at pH 8.0, 500 mM NaCl, 0.5% Nonidet P-40, 1 mM dithiothreitol, and protease inhibitor tablets from Roche Applied Science), and immunoprecipitated with 20 μg anti-FLAG (Sigma-aldrich, CA, US) overnight at 4 °C. The beads were washed three times with the lysis buffer and eluted in SDS sample buffer. The eluted immunocomplexes were separated by SDS-PAGE, followed by western blotting with indicated antibodies according to the standard procedures. For detecting endogenous protein interactions, cells were lysed in 500 μL of lysis buffer and immunoprecipitated with indicated antibody or control serum (Santa Cruz Biotechnology, CA, US). After extensive washing with the lysis buffer, the immunoprecipitates were resolved by SDS-PAGE, followed by western blot analysis.

### Quantitative reverse transcrition PCR (qRT-PCR)

Total RNA was extracted from the cells (1 × 10^6^ cells) using Trizol plus kit (TaKaRa, Japan). First-strand cDNA synthesis was performed using Promega kit (CA, US). Synthesized cDNA was used for qRT-PCR analysis using Lightcycler (Roche, Switzerland) based on the manufacturer’s instructions. β-actin was used as an internal control. All samples were amplified in duplicates. Relative changes in the number of transcripts in each sample were determined with β-actin normalization of mRNA levels.

### Chromatin Immunoprecipitation (ChIP) and re-ChIP

For ChIP assay, 2 × 10^5^ cells were sonicated and centrifuged. The supernatants were collected and incubated overnight with indicated antibodies and Protein G magnetic beads. The beads were washed, and the precipitated chromatin complexes were collected, purified, and de-crosslinked, followed by incubation at 95 °C for 10 min. The precipitated DNA fragments were measured by RT-PCR assay. For re-ChIP, complexes were eluted from the primary immunoprecipitation by incubation with 10 mM DTT at 37 °C for 30 min and diluted in re-ChIP buffer followed by re-immunoprecipitation with the secondary antibodies.

### ChIP-seq and ChIP-qPCR analysis

ChIP was performed as previously described [12]. For ChIP-seq, 200 μg of chromatin was incubated with 10 μg of antibody overnight. The precipitates were washed and eluted at 65 °C. Chromatin was decrosslinked and DNA was purified. The enrichment of target sequences was checked by qRT-PCR. For ChIP-qPCR analysis, 100 μg of chromatin and 5 μg of Notch1 or TAZ antibody were used. The amounts of immunoprecipitated DNA were determined by quantitative real-time PCR.

### Flow cytometry assay

Anti-CD3-FITC and anti-CD8-PE antibodies were used for intracellular staining. Cells were washed twice with Hank’s balanced salt solution (HBSS), then fixed and permeabilized in Fix/Perm buffer according to the manufacturer’s instructions for 30 min. 3 × 10^4^ cells were washed twice with permeabilization buffer and then incubated with appropriate antibodies at 4 °C for 30 min in the dark. Unbound antibodies were removed by washing twice with permeabilization buffer. Flow cytometry analyses were performed on a three-color fluorescence FACSCalibur flow cytometer using Cell Quest software (Becton-Dickinson, CA, USA).

### T cell-mediated tumor cell killing assay, T cell apoptosis assays, and T cell IL-2 production assay

CD3 + CD8+ cytotoxic T lymphocytes (CTL) were purified and cocultured with A549 cells transfected with LDHA shRNA in the presence or absence of lactate for 7 days. To study the involvement of T cell-mediated tumor cell killing, 2 × 10^5^ cells were activated by incubation with anti-CD3 antibody (100 ng/mL) and IL-2 (10 ng/mL). Activated cytotoxic T-cell–directed cytotoxicity toward 2 × 10^5^ A549 cells was performed by the method as described previously [17]. Percentage of cytotoxicity was calculated using the following formula: % cytotoxicity = (lysis from effector-target mixture-lysis from effector only) − spontaneous lysis/maximum lysis − spontaneous lysis × 100. T cell apoptosis was measured using the Caspase 3/7 assay (Promega, CA). T cell IL-2 levels were measured using the IL-2 Human ELISA kit (Invitrogen, CA).

### Glucose uptake, lactate production, pyruvate kinase activity, and extracellular acidification rate (ECAR)

To measure glucose uptake, 2 × 10^5^ cells were incubated with a fluorescent D-glucose derivative, 2-[N-(7-nitrobenz-2-oxa-1,3-diazol-4-yl)amino]-2- deoxy-D-glucose (2-NBDG; Invitrogen, CA) for 10 min. The fluorescence intensity of 2-NBDG was measured using a microplate reader SynergyH4 (BioTeK) and normalized to that of 2-NBDG by total protein amount. Lactate production (Lactate Assay Kit, CA, US) and pyruvate kinase activity (Pyruvate Activity Assay Kit, CA, US) were measured as described by the manufacturer (BioVision). ECAR was measured using extracellular flux analyzer (XF96) analyzer (Seahorse Bioscience, CA, US) as described by the manufacturer.

### Transcriptome sequencing (RNA-seq)

A minimum of 3 mg of total RNA was oligo (dT) selected using the Dynabeads mRNA purification kit (Invitrogen, CA, US). The mRNAs isolated from total RNA were fragmented into short fragments with fragmentation buffer (Ambion, CA, US). Double-stranded cDNA was synthesized with these short fragments as templates. The cDNA was end-repaired, ligated to Illumina adapters, size selected on agarose gel, and PCR amplified. The cDNA library was sequenced on an Illumina HiSeq 2000 sequencing platform. The gene expression levels for each transcript were estimated as the number of reads per kilobase of exon model per million mapped reads using uniquely mapped reads in exonic regions. A gene is considered significantly differentially expressed if its expression differs between any two samples with the fold change > 2 and the *P* value < 0.05 as calculated by Cufflinks.

### RNA-Seq read counting

The first step consists of de-multiplexing the reads by recognizing specific adapter sequences to assign each read to the corresponding sample (three samples were pooled per flow cell lane). From 100 to 240 million paired-end reads were obtained per flow cell lane, corresponding to 27 to 91 million reads for each cDNA library. These paired-end reads were then mapped back to the reference transcriptome, using Bos taurus known transcripts recorded in the Ensembl database v.6. This set contains 27,663 transcript sequences assigned to 21,734 known genes and pseudogenes. Paired-end reads located exactly on the same transcript were selected and counted.

### ^18F^FDG PET-CT and immunohistochemistry assay

We retrospectively reviewed twenty-three newly diagnosed patients with NSCLC from the PET-CT database of the Department of Nuclear Medicine at The First Affiliated Hospital of Guangzhou Medical University from October 2019 to August 2020. The protocol was approved by Ethics Committee. All patients were selected consecutively. The inclusion criteria for the study were as follows: (1) new diagnosis of NSCLC, (2) available pathology reports, (3) lesion sampled by surgical resection. ^18F^FDG PET-CT scan was conducted using a Siemens whole-body PET-CT scanner (Siemens, Germany). The precise maximal standard uptake values (SUVmax) of ^18F^FDG were independently measured on PET-CT images by two experienced nuclear medicine physicians. Immunohistochemistry (IHC) of formalin-fixed paraffin-embedded samples was performed as described previously [7]. Briefly, the formalin-fixed paraffin sections were deparaffinized, rehydrated, and pretreated with 3% H_2_O_2_ for 20 min. The antibody-binding epitopes of the antigens were retrieved by microwave treatment, and the sections were then preincubated with 10% goat serum to block nonspecific binding. Rabbit anti-Notch1 (Santa Cruz Biotechnology) and rabbit anti-TAZ (Santa Cruz Biotechnology) were used as the primary antibodies. The specimens were incubated with the primary antibodies for 1 hr at room temperature, followed by the addition of anti-rabbit secondary antibody and streptavidin-horseradish peroxidase. The score of Notch1 and TAZ was generated by multiplying the percentage of stained cells (0–100%) by the intensity of the staining (low, 1+; medium, 2+; strong, 3+). H-score > 150 was considered strong expression of Notch1 and TAZ.

### Statistical analysis

Data processing was performed using the Statistical Package (SPSS v.15.0; USA). All intergroup differences were analyzed using the Mann–Whitney test or Wilcoxon test. All results are presented as the mean ± standard error of the mean (SEM) or standard deviation (SD). *P*-values <0.05 were considered statistically significant.

## Results

### Notch1 stimulates glycolysis in lung cancer cells

**Fig. 1:**
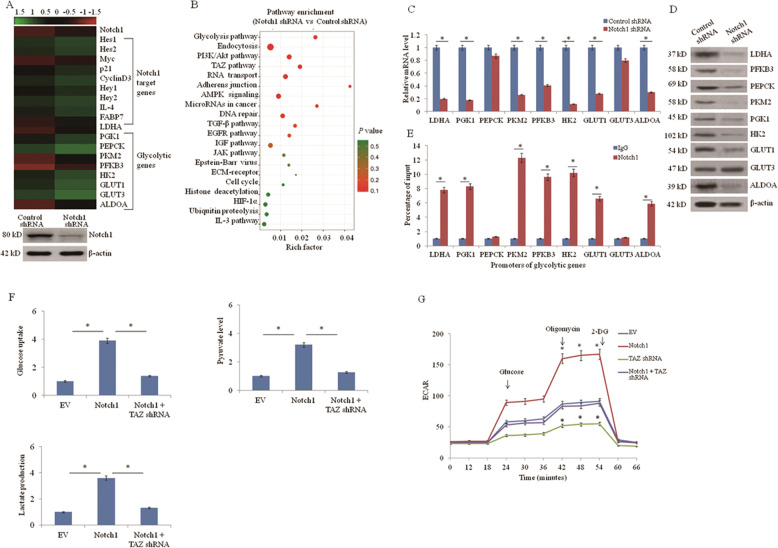
Notch1 stimulates glycolysis in lung cancer cells. **a** Heatmap of known Notch1 target genes and glycolytic genes identified by RNA-seq using A549 cells stably transfected with Notch1 short hairpin RNA (shRNA) or control shRNA. Western blot shows the knockdown of Notch1 expression. **b** KEGG pathway analysis of genes differentially expressed between A549 cells stably transfected with Notch1 shRNA or control shRNA. **c**, **d** The mRNA and protein expression of glycolytic genes in A549 cells stably transfected with Notch1 shRNA or control shRNA were examined by qRT-PCR (**c**) and western blot (**d**) respectively. **e** ChIP analysis of Notch1 occupancy on promoters of glycolytic genes in A549 cells. IgG: normal serum. The different number after each gene represents the regions containing different Notch1-binding sites. The graph shows the percentage of input. **f**, **g** A549 cells were transfected with empty vector (EV), Notch1 intracellular domain (ICD), or TAZ shRNA. Glucose uptake, pyruvate level, lactate production level (**f**), and extracellular acidification rate (ECAR) (**g**) were examined. *P < 0.05.

To identify Notch1 downstream effectors, we performed RNA sequencing (RNA-seq) using Notch1 knockdown with shRNA in A549 cell line or control cell line. Notch1 regulated the expression of over 1,000 genes, including previously reported Notch1-regulated genes (accession number GEO: GSE137106) (Fig. 1a). KEGG (Kyoto Encyclopedia of Genes and Genomes) analysis showed that the top 20 enriched pathways included the glycolysis pathway (Fig. 1b). Next, we investigated the effect of Notch1 on the glycolysis pathway. The expression of glycolysis-associated genes LDHA, PFKB3, PKM2, PGK1, HK2, GLUT1, ALDOA, PEPCK, and GLUT3 were measured by qRT-PCR and western blot assay (Fig. 1c, d). ChIP assay showed the Notch1 occupancy on promoters of glycolytic genes in A549 cells (Fig. 1e). Notch1 shRNA significantly decreased the expression of LDHA, PFKB3, PKM2, PGK1, HK2, GLUT1, and ALDOA at the mRNA and protein levels. Notch1 increased glucose uptake, pyruvate level, and lactate production in A549 cells. These effects were reversed by TAZ knockdown (Fig. 1f). Notch1 also increased the ECAR. Again, TAZ shRNA abolished the ability of Notch1 to regulate these effects, indicating that Notch1 promotes glycolytic gene expression via TAZ (Fig. 1g).

### Notch1 promotes glycolytic gene expression through functional interaction with histone acetyltransferases p300 and pCAF

**Fig. 2:**
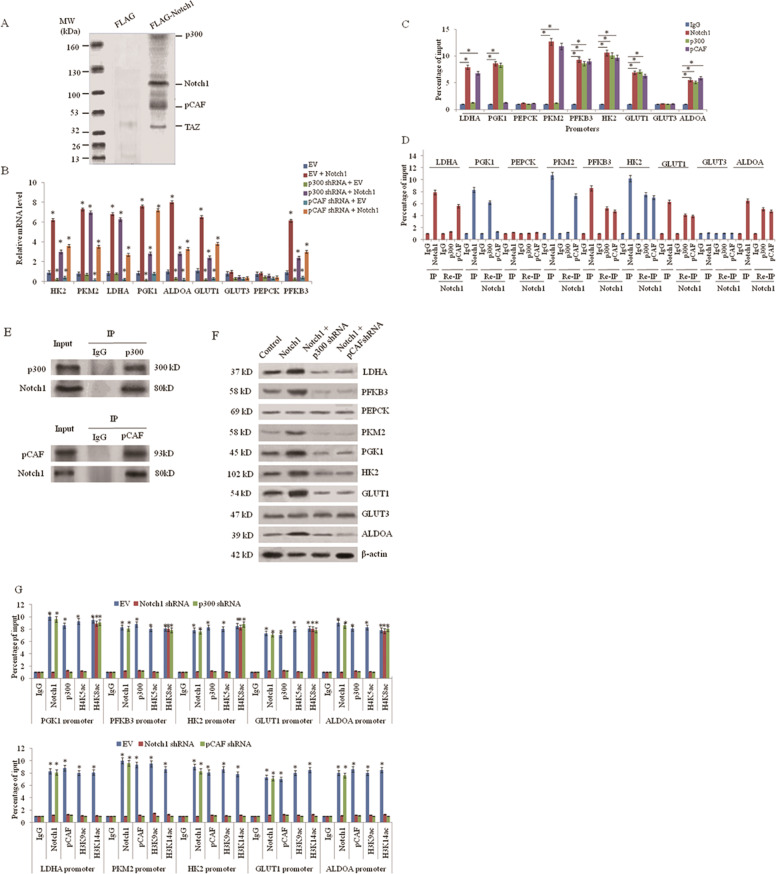
Notch1 promotes glycolytic gene expression through functional interaction with p300 and pCAF. **a** Cellular extracts from A549 cells stably expressing FLAG (control) or FLAG-Notch1 were immunopurified with anti-FLAG affinity columns and eluted with FLAG peptide. The eluates were resolved by SDS-PAGE and silver stained. The differential protein bands were retrieved and analyzed by mass spectrometry. **b** A549 cells were transfected with Notch1 ICD, p300 shRNA, pCAF shRNA, or empty vector (EV). Glycolytic gene expression was measured using qRT-PCR. *P < 0.05. **c** ChIP analysis of Notch1, p300, and pCAF occupancy on glycolytic gene promoters in A549 cells. The graph shows the percentage of input. **d** Re-ChIP analysis of the occupancy of Notch1 and p300 or pCAF on the glycolytic gene promoters in A549 cells. **e** A549 cells were immunoprecipitated with anti-p300, anti-pCAF, or normal IgG, and the precipitates were analyzed by immunoblot with the indicated antibodies. IP immunoprecipitation. **f** A549 cells stably transfected with Notch1 were co-transfected p300 shRNA or pCAF shRNA. The protein expression of glycolytic genes was examined using western blot assay. **g** Notch1, p300, pCAF, and histone H3 and H4 acetylation occupancy on the promoters of indicated glycolytic genes in A549 cells transfected with Notch1 shRNA, p300 shRNA or pCAF shRNA was examined using ChIP assay.

Interaction of transcription factors with histone-modifying enzymes is required to regulate gene transcription, and histone acetylation regulated by histone acetyltransferases such as p300 and pCAF is often associated with active transcription [18]. To investigate how Notch1 stimulates glycolytic gene transcription, we used Co-IP combined with mass spectrometry to identify its interaction partners. Besides the previously reported Notch1-interacting protein TAZ, we identified two histone acetyltransferases, p300, and pCAF, as potential Notch1 interaction partners (Fig. 2a). Peptides identified by mass spectrometry analysis of Notch1-containing protein complex that were eluted from A549 cells stably transfected with FLAG-tagged Notch1 were shown in Supplementary Table S1. P300 and pCAF act cooperatively to mediate transcriptional activation from chromatin templates by Notch intracellular domain in vitro [19]. Next, we tested whether Notch1 regulates glycolytic gene transcription via p300 and pCAF. p300 shRNA or pCAF shRNA decreased mRNA level of HK2, PKM2, LDHA, PGK1, ALDOA, GLUT1, and PFKB3, but not the other glycolytic genes GLUT3 and PEPCK. Moreover, p300/pCAF knockdown by shRNA greatly attenuated the ability of Notch1 to promote mRNA expression of glycolytic genes (Fig. 2b). ChIP assay showed that Notch1 regulates glycolytic gene transcription. p300 was recruited to the Notch1 binding sites of PGK1, PFKB3, HK2, GLUT1, and ALDOA promoters, and pCAF was recruited to those of LDHA, PKM2, PFKB3, HK2, GLUT1, and ALDOA promoters (Fig. 2c). Re-ChIP assay verified that Notch1 associated with p300 or pCAF on the corresponding binding sites of glycolytic genes (Fig. 2d). Co-IP of endogenous proteins verified the interaction between Notch1 and p300/pCAF (Fig. 2e). p300 shRNA or pCAF shRNA significantly attenuated the ability of Notch1 to increase the protein expression of LDHA, PFKB3, PKM2, PGK1, HK2, GLUT1, and ALDOA (Fig. 2f). Next, we investigate how Notch1 regulates glycolytic gene transcription through p300 and pCAF, which acetylate histone H3 and H4. p300 knockdown significantly decreased recruitment of H4K5ac, but not H4K8ac, to the Notch1/p300 binding sites, and pCAF knockdown led to a marked reduction of recruitment of H3K9ac, but not H3K14ac, to the Notch1/pCAF binding sites (Fig. 2g).

### Notch1 signaling forms a positive feedback loop with TAZ

**Fig. 3:**
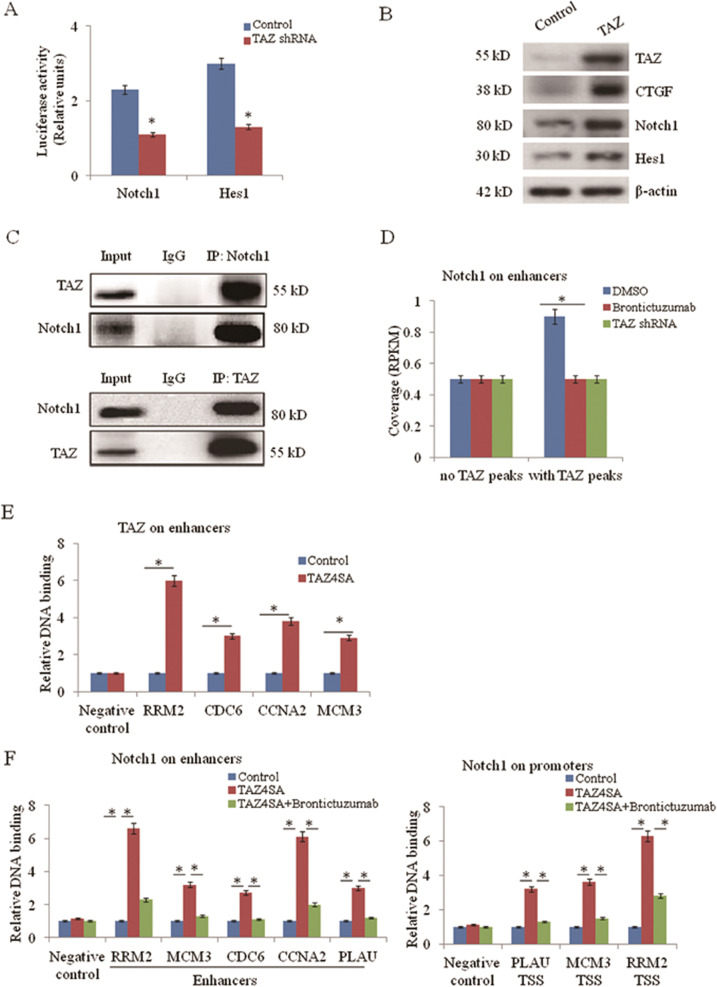
Notch1 signaling forms a positive feedback loop with TAZ. **a** The reporter activity of Notch1 and Hes1 in A549 cells transfected with TAZ was measured by luciferase reporter assay. *P < 0.05. Relative luciferase activity was performed to identify Notch responsive region in the Hes1 promoter. **b** The protein levels of Notch1 and Hes1 induced by TAZ overexpression in A549 cells were examined by western blot assay. **c** Notch1 ICD associated with endogenous TAZ in A549 cells. Immunoprecipitation (IP) was performed using antibodies of Notch1 ICD and TAZ, and coprecipitated protein was analyzed by western blot assay. **d** Comparison of Notch1 ChIP-seq signal (expressed as normalized read density, RPKM) in active enhancers with or without TAZ peaks in A549 cells treated with DMSO, Brontictuzumab (2 μm for 5 h), or cells transfected with TAZ shRNA. *P < 0.05. **e** TAZ binding at enhanc**e**rs of TAZ target genes in TAZ^4SA^ overexpressing A549 cells by ChIP-qPCR analysis. DNA enrichment was calculated and presented as fold vs control cells. **f** ChIP-qPCR analysis showed Notch1 binding on enhancers and promoters (TSS, transcription start site) of TAZ targets upon TAZ^4SA^ overexpressing in A549 cells, but not in the presence of Brontictuzumab (2 μm for 5 h).

Because knockdown of TAZ reversed the ability of Notch1 to increase aerobic glycolysis in lung cancer, we next asked whether hippo/TAZ signaling interactions with Notch1 signaling promotes aerobic glycolysis. Knocking down TAZ led to decreased reporter activities of Notch1 and its target gene Hes1 in the A549 cells (Fig. 3a). ChIP assay further confirmed that TAZ binds directly to the Notch1 promoter (Supplementary Fig. S1). In addition, we found that overexpressing TAZ in A549 cells led to upregulated expressions of CTGF, Notch1, and Hes1 (Fig. 3b). It was reported that Notch1 might directly bind to TAZ [20], but not furtherly confirmed. So we hypothesized that Notch1 might interact with TAZ. To elucidate how Notch1 interacts with TAZ, we conducted Co-IP experiments by immunoprecipitating either Notch1 or TAZ. The results showed that TAZ was pulled down by Notch1 immunoprecipitation in A549 cells, and Notch1 was also pulled down by TAZ immunoprecipitation (Fig. 3c). We next investigated the mechanism of interaction between Notch1 and TAZ. ChIP-seq analysis was performed to compare Notch1 and TAZ binding to chromatin. Genes bounded by TAZ and Notch1 in A549 cells are shown in Supplementary Table S2. The results showed that Notch1 coverage was higher on enhancers containing TAZ binding sites when compared to active enhancers not occupied by TAZ. Notch1 inhibitor Brontictuzumab induced a loss of Notch1 from TAZ-occupied enhancers compared to active enhancers without TAZ binding sites (Fig. 3d). Thus, the presence of TAZ peaks defines enhancers enriched of Notch1 and highly sensitive to Notch1 inhibitors on the genome-wide scale. We validated the functional interdependency between Notch1 and TAZ proteins by overexpressing TAZ^4SA^ (active mutation of TAZ) in A549 cells. By ChIP assay, exogenous TAZ is recruited as its cognate chromatin sites (Fig. 3e). In turn, this leads to Notch1 recruitment at the same enhancer sites and associated promoters (Fig. 3f).

### TAZ regulates Jagged1 in a TEAD1-dependent manner

**Fig. 4:**
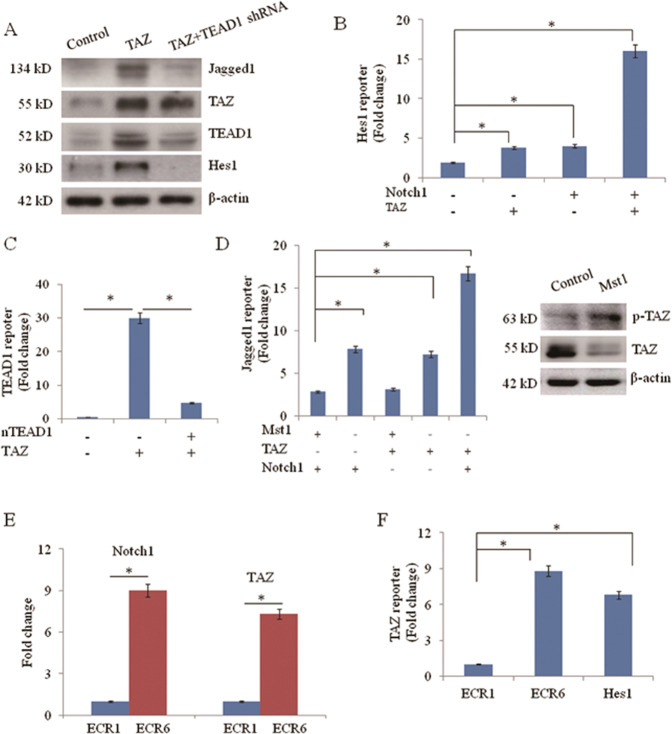
TAZ regulates Jagged1 in a TEAD1-dependent manner. **a** Protein analysis of Jagged1, TAZ, TEAD1, and Hes1 in A549 cell lysates after transfected with TAZ and concomitant silencing of TEAD1 by shRNA. **b** Hes1 reporter assay in the presence (+) or absence (−) of Notch1 ICD, or TAZ, *N* = 3. Results of luciferase reporter assays are shown. *P < 0.05. **c** TEAD1-reporter luciferase assay in the presence (+) or absence (−) of TAZ or nTEAD1, *N* = 3. **d** Jagged1 luciferase reporter assay in the presence (+) or absence (−) of Notch1 ICD, TAZ, or Mst1, *N* = 3. *P < 0.05. Cells were transfected with or without MST1, phosphorylated TAZ and TAZ were analyzed by western blot assay. **e** ChIP assay for Notch1 or TAZ in A549 cells transfected with Notch1, TAZ, and plasmid of either Jagged1-ECR1 or ECR6. Data are presented as fold enrichment over an IgG ChIP performed with the same samples. **f** ChIP assay for TAZ in A549 cells at Jagged1-ECR1, ECR6, and Hes1 promoter. *N* = 3. *P < 0.05.

TAZ gains its target gene-specificity through a panel of transcription factors, such as TEAD family members [8]. To identify the transcription factors responsible for TAZ-induced Jagged1 expression, Jagged1 transcript levels were examined after shRNA-mediated inhibition of TEAD1 in A549 cells. TEAD1-specific shRNA led to a comparable reduction in transcript and protein levels of Jagged1 and Hes1. Interestingly, overexpression of TAZ levels also increased TEAD1 levels, indicating that TAZ positively regulates TEAD1. Overexpression of TAZ and concomitant inhibition of TEAD1 revealed that silencing of TEAD1 abolished TEAD-induced expression of Jagged1 and Hes-1 (Fig. 4a). Hes1 is a canonical, direct target of Notch1 signaling. Hes1 promoter mutant report construct was generated using site-directed mutagenesis (Supplementary Table S3). Notch1 ICD activation of this Hes1 reporter is significantly enhanced by the addition of TAZ (Fig. 4b). Dominant-negative form of TEAD1 (nTEAD1) inhibited TAZ activation of a control reporter construct containing TEAD1 binding sites (Fig. 4c). As previously reported, Notch1 intracellular domain (ICD) activates a reporter construct containing Jagged1 upstream of a synthetic minimal promoter driving luciferase expression [2, 3]. Interestingly, TAZ is also able to activate this reporter, and the combination of Notch1 ICD and TAZ generates significantly more activity than either Notch1 or TAZ alone. TAZ activity is regulated by the Hippo kinase cascade (Mst and Lats), which control TAZ nuclear localization [8]. As expected, co-transfection of a construct encoding Mst1 abrogates the ability of TAZ to activate Jagged1, either alone or in combination with Notch1. Western blot showed that upon MST1 exogenous overexpression, TAZ phosphorylation was increased (Fig. 4d). These results indicate that the Hippo kinase Mst1 inhibits Notch signaling by inhibiting the expression of Jagged1 that is activated by TAZ. Comparative sequence analysis of the Jagged1 genomic locus identified 15 evolutionarily conserved regions (ECRs). A single Jagged1 ECR, located within Intron 2, displayed Notch responsive activation and this 617 bp element was denoted “ECR6” [21]. ChIP assay showed that Notch1 and TAZ-occupied ECR6, but not ECR1 (upstream Jagged1 enhancer which is not activated by Notch1) (Fig. 4e). ChIP for endogenous TAZ in A549 cells showed co-occupancy of the endogenous Hes1 and ECR6, but not ECR1 (Fig. 4f).

### Notch1/TAZ axis regulates glycolysis and tumor growth in vitro and in vivo

**Fig. 5:**
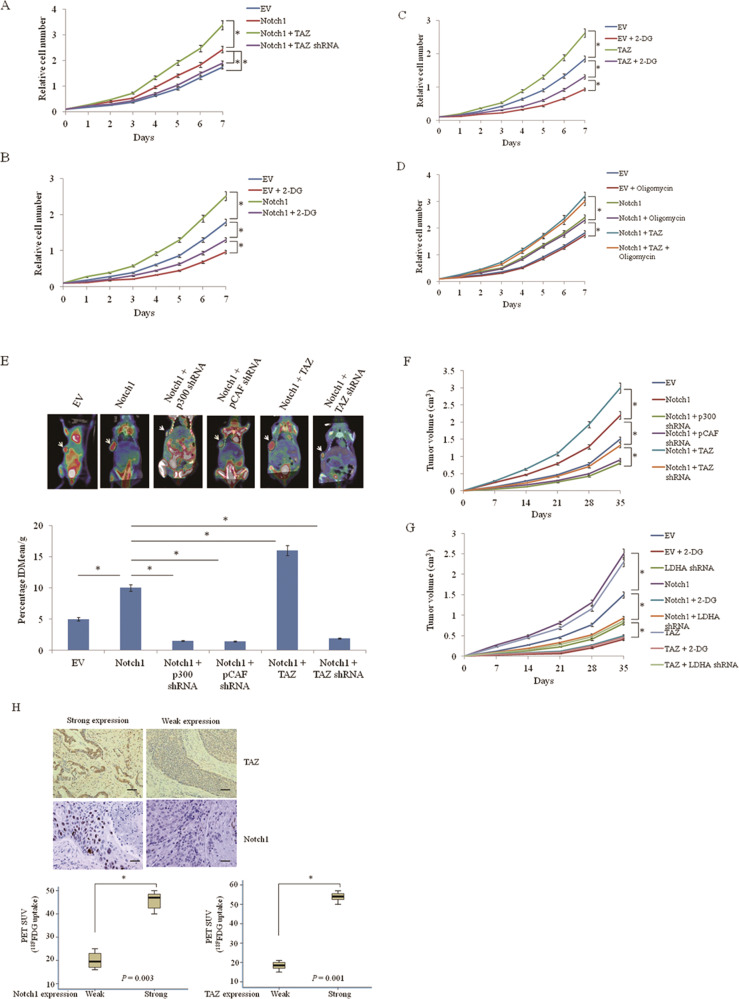
Aerobic glycolysis is critical for Notch1/TAZ axis modulation of lung cancer growth in vitro and in vivo. **a** The proliferation curve of A549 cells transfected with Notch1, TAZ, TAZ shRNA or empty vector. Cell proliferation was determined by the CCK-8 Kit. *P < 0.05. **b** The proliferation curve of A549 cells transfected with Notch1 or empty vector, treated with 2.5 mM 2-DG as indicated. Cell proliferation was determined by the CCK-8 Kit. *P < 0.05. **c** The proliferation curve of A549 cells transfected with TAZ or empty vector, treated with 2.5 mM 2-DG as indicated. Cell proliferation was determined by the CCK-8 Kit. *P < 0.05. **d** The proliferation curve of A549 cells transfected with Notch1, TAZ or empty vector, treated with 0.1 mM Oligomycin in normal culture medium (containing 25 mM glucose) as indicated. Cell proliferation was determined by the CCK-8 Kit. *P < 0.05. **e** MicroPET-CT imaging of nude mice to determine FDG uptake in mice with subcutaneous xenograft lung cancer model established with A549 cells transfected with empty vector, p300 shRNA, pCAF shRNA, Notch1, TAZ, Notch1 shRNA, or TAZ shRNA. Representative ^18F^FDG microPET images are shown with arrowheads indicating xenografted lung cancers at treatment end (day 35). Quantification of ^18F^FDG uptake in tumors is shown as %IDmean/g. *P < 0.05. **f** Xenograft tumors were established as in (**b**) and the growth curve was plotted. *P < 0.05. **g** A549 cells stably expressing Notch1, TAZ, or LDHA shRNA were subcutaneously injected into nude mice. 2-DG was used as indicated. The growth curve was plotted. *P < 0.05. **h** Representative expression of Notch1 and TAZ by immunohistochemistry assay of 23 lung cancer patients. The correlation of glucose uptake with Notch1 or TAZ expression was determined using the Mann–Whitney *U* test. Scale bar = 50 μm. Original magnification: ×100. *P < 0.05.

We tested whether Notch1/TAZ axis regulates lung cancer cell proliferation in cultured cells. Notch1 increased lung cancer cell proliferation. This effect was enhanced by TAZ co-transfection, while TAZ knockout almost abolished Notch1-stimulated cancer cell proliferation (Fig. 5a, Supplementary Fig. S2A). We then examined whether glycolysis plays a role in Notch1/TAZ axis-mediated regulation of lung cancer cell proliferation. Tumor cells tend to avoid mitochondrial activity and oxidative phosphorylation (OXPHOS) and prefer glycolysis for energy production [22]. As expected, glycolytic inhibitors 2-deoxy-D-glucose (2-DG) inhibited cancer cell proliferation. Importantly, 2-DG, but not the OXPHOS inhibitor oligomycin greatly reduced the ability of Notch1 and TAZ to promote cancer cell proliferation (Fig. 5b–d, Supplementary Fig. S2B–D). These data suggest that increased glycolysis by Notch1 promotes cancer cell proliferation other than OXPHOS. To examine the effects of Notch1/TAZ axis on glycolysis in vivo, we used ^18F^FDG micro-PET (positron emission tomography) scans to measure glucose uptake in tumor xenografts in nude mice. Notch1 greatly increased glucose uptake while knockdown of p300 or pCAF greatly reduced the ability of Notch1 to increase glucose uptake. Glucose uptake increased by Notch1 was enhanced in the presence of TAZ and reversed by TAZ knockdown (Fig. 5e). These data suggest that TAZ regulates glycolysis via interaction with Notch1 and that Notch1 modulates glycolysis through p300 and pCAF in nude mice. The tumors with higher glycolysis grew faster (Fig. 5f). Next, we tested whether glycolysis plays a role in Notch1/TAZ axis-mediated regulation of tumor growth in nude mice. As expected, the glycolytic inhibitor 2-DG and knockdown of LDHA, the enzyme that catalyzes the final step of glycolysis, inhibited tumor growth (Fig. 5g). The results indicate that glycolysis mediated by Notch1/TAZ axis is critical for lung cancer growth. To examine the physiological relevance of Notch1/TAZ axis in glycolysis, the correlation of glucose uptake with Notch1 or TAZ expression was determined using the Mann-Whitney *U* test. Lung cancer patients who had increased glucose uptake assessed by ^18F^FDG PET scans showed increased Notch1 expression and increased TAZ expression (Fig. 5h).

### Notch1 signaling induced lactate inhibits T and NK cell activation

**Fig. 6:**
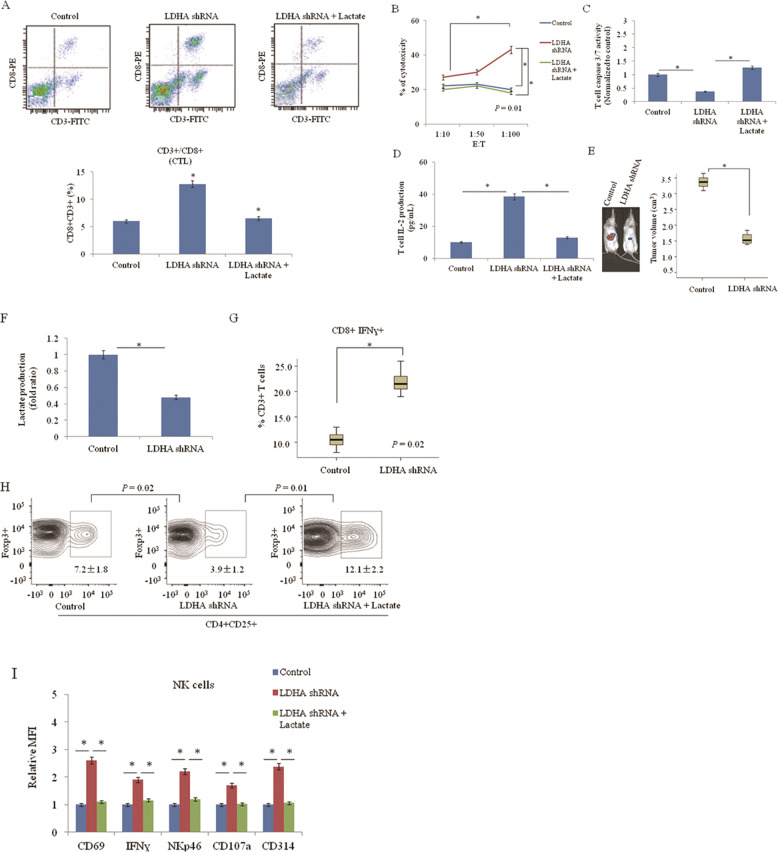
Notch1 signaling induced lactate inhibits cytotoxic T cell activity. **a** Flow cytometric analysis of cytotoxic T lymphocytes (CTL) in coculture of primary T cells and A549 cells transfected with LDHA shRNA with or without 10 mmol/L lactate treatment for 96 h. *P < 0.05. **b** CTL cells isolated from untreated lung cancer patients were cultured in vitro for 3 days and 7 days, respectively. A549 cells were transfected with LDHA shRNA. CTL cells were incubated with A549 cells with or without lactate for 96 h at different E:T ratio (1:10, 1:50, and 1:100). Cytotoxicity of CTL cells toward lung cancer cells was determined by LDH release assay (*P < 0.001, when effector cells were stimulated with lactate in comparison to unstimulated cells). **c**, **d** T cell Caspase 3/7 activities (**c**) and IL-2 level (**d**) were measured in CTL cells incubated with A549 cells transfected with LDHA shRNA with or without 10 mmol/L lactate treatment. *N* = 3. *P < 0.05. **e** Representative images of tumor growth of LDHA shRNA-expressing A549 xenograft models in BALB/c mice by bioluminescence imaging. Tumors were measured every five days and dissected at day 35, and tumor size (cm^3^) is shown in a box-and-whisker plot. *N* = 8 mice per group. *P < 0.05. **f** Lactate production in LDHA knockdown A549 xenograft tumors. *P < 0.05. **g** Intracellular cytokine staining of IFNɣ in CD3 + CD8+ T-cell populations in LDHA knockdown-A549 xenograft tumors. *P* < 0.05, two-way ANOVA. *P < 0.05. **h** Flow cytometry analysis of CD4 + CA25 + Foxp3+ T regulatory (Treg) cells in LDHA knockdown-A549 xenograft tumors. Treg cells were treated with or without 10 mmol/L lactate for 96 h. **i** Flow cytometry analysis of activation markers of CD69, IFNɣ, CD107a, CD314, and NKp46 in NK cells in LDHA knockdown-A549 xenograft tumors. NK cells were treated with or without 10 mmol/L lactate for 96 h. Relative MFI, mean fluorescence intensity.

**Fig. 7:**
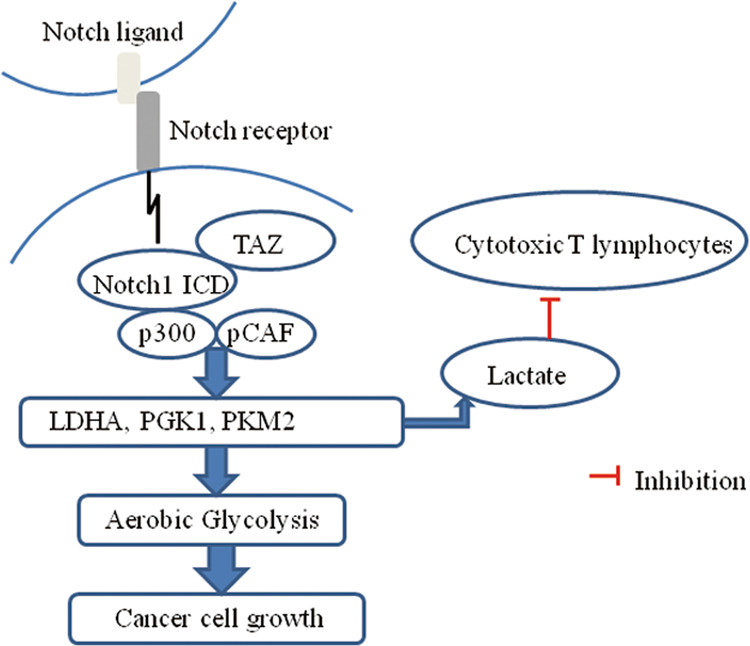
Schematic diagram illustrating the mechanism of Notch1/TAZ axis involved in aerobic glycolysis and immune escape in lung cancer. Notch1 forms a positive feedback loop with TAZ and promotes glycolytic gene expressions through interaction with p300 and pCAF. Increased levels of extracellular lactate via Notch1/TAZ loop inhibits cytotoxic T-cell activity, which contributes to lung cancer invasion.

On the basis of our findings that Notch1/TAZ axis induces glycolysis, which leads to the accumulation of glycolytic metabolites in tumor cells and increased extracellular level of lactate (Fig. 1e) and a previous report demonstrating that extracellular lactate inhibits cytotoxic T-cell activity in breast cancer [23], we next asked whether extracellular lactate modulates cytotoxic T cell-related functions in lung cancer cells. To this end, we analyzed cytotoxic T-cell activity with or without the addition of lactate by using activated primary human T cells. Coculture of T cells and A549 cells transfected with LDHA shRNA had increased CTL population compared with control cells (Fig. 6a), while the addition of lactate reversed the effect of increased CTLs by LDHA knockdown. Consistently, LDHA shRNA increases the cytotoxic potential of T cells toward cancer cells in a T-cell–mediated tumor cell killing assay. The addition of lactate also reduced the number of CTLs in A549 cells transfected with LDHA shRNA (Fig. 6b). Accordingly, LDHA knockout in A549 cells suppressed T cell apoptosis and this was reversed by the addition of lactate (Fig. 6c). T cell IL-2 level was enhanced when glycolytic gene LDHA was inhibited and reduced after addition of lactate (Fig. 6d). These data are consistent with a model in which Notch1 pathway promotes immune evasion through aerobic glycolysis. To further investigate whether this phenomenon occurs in vivo, we examined tumor growth, lactate production, the population of activated cytotoxic T-cell, T cell caspase 3/7 activity, and T cell IL-2 production in A549 xenograft tumors. In line with the results from in vitro experiments, LDHA knockdown inhibited tumor growth, reduced lactate level, and enhanced cytotoxic T cell activity (Fig. 6e–g). CD4 + CA25 + Foxp3+ T regulatory (Treg) cells were isolated from A549 xenograft tumors with or without LDHA knockout. Treg cells were treated in presence of lactate treatment. Flow cytometry analysis revealed a decrease population of Treg cells in LDHA knockout xenograft tumor, while exogenous lactate significantly increased the number of Treg cells (Fig. 6h). Significant increases of CD69, IFNɣ, CD107a, CD314, and NKp46 in tumor infiltrated NK cells in LDHA knockout xenograft tumor. The addition of lactate decreased these activated markers in tumor infiltrated NK cells (Fig. 6i). The results suggest that increased levels of extracellular lactate via Notch1/TAZ axis inhibits cytotoxic T-cell activity and the activation of tumor infiltrated NK cells, which may contribute to the invasive nature of lung cancer cells. Together the results suggest that interaction between Notch1 and TAZ promotes aerobic glycolysis and immune escape in lung cancer (Fig. 7).

## Discussion

The metabolic phenotype of lung cancer cells is characterized by increased glucose uptake and glycolytic activity [24, 25]. This metabolic reprogramming supports lung cancer cells with rapid proliferation and survival. Our results show that Notch1/TAZ axis mediates aerobic glycolysis. Moreover, Notch1 directly promotes the expression of many key glycolytic genes that facilitates aerobic glycolysis and tumor growth.

Notch1 is overexpressed and promotes tumor growth in lung adenocarcinoma [3, 26]. Our data show that Notch1 regulates Notch1 knockdown lung cancer cells regulates glycolytic gene expression, glucose uptake, and the level of lactate, a metabolite that can facilitate tumor growth, indicating that Notch1 is a key transcription factor for the regulation of aerobic glycolysis. Notch1 signaling increases the rate of glycolysis through the upregulation of glycolysis-related gene expressions such as GLUT1 and GLUT3 [27, 28]. The function of Notch1 in glycolysis in lung adenocarcinoma at least partly explains the inhibited expression of glycolytic genes induced by Notch1 knockdown. However, we cannot exclude the possibility that other genes regulated by Notch1 may also be responsible for these defects.

Potentiation of glycolytic gene transcription by Notch1 is mediated mainly through histone acetyltransferases p300 and p300/CBP-associated factor (pCAF) [19]. It is well established that transcription factors can orchestrate the recruitment of histone-modifying enzymes to specific sets of target genes [18]. The exact roles of p300 and pCAF in stimulating glycolytic gene expression remain to be investigated. Our study indicates that p300-mediated H4K5ac and pCAF-mediated H3K9ac are essential for the Notch1 regulation of glycolytic genes (LDHA, PKM2, PFKB3, PKM2, HK2, GLUT1, and ALDOA). pCAF is a co-factor shared between p53 and HIF-1 in the regulation of glycolytic production by modulating SCO2 and TIGAR gene expression [29]. p300 functions as a lysine 2-hyroxyisobutyryltransferase to regulate glycolysis in response to nutritional cues. P300-deficient cells have impaired glycolysis and are hypersensitive to glucose-depletion-induced cell death [30]. The transcriptional activity of p300 requires acetyl coenzyme A, indicating that it functions as a histone acetyltransferase when mediating Notch intracellular domain function. pCAF is unable to promote transcription on its own but enhances Notch intracellular domain-mediated transcription from chromatin in conjunction with p300 [19].

Notch and Hippo/TAZ signaling functionally interact to promote tumor progression [31, 32]. Modulation of Hippo signaling has been reported to alter Notch target gene expression. Gao et al. showed that genetic Mst1/2 knockout increased YAP and Hes1 expression [33]. The deletion of the Hippo effector molecules YAP and TAZ disrupt Notch signaling. TAZ is normally recruited to a tissue-specific Jagged1 enhancer by directly interacting with the Notch intracellular domain. Thus, Hippo signaling can modulate Notch signaling outputs, and components of the Hippo and Notch pathways physically interact [34]. Accordingly, our results show that TAZ promotes the expression of Notch1 and Notch1 stabilizes TAZ protein, indicating a potent positive feedback loop between TAZ and Notch signaling in lung cancer that may orchestrate aerobic glycolysis. Notch1 transcriptional activity is increased in the presence of TAZ and the activation is TEAD1 dependent. Both Notch1 and TAZ are overexpressed in lung adenocarcinoma and associated with poor prognosis in lung adenocarcinoma patients. Notch1 stimulates glycolysis in lung cancer and TAZ promotes this effect via interaction. Lung cancer cells use aerobic glycolysis more than oxidative respiration (OR) for metabolic programming. The glycolytic inhibitor 2-DG, but not the OXPHOS inhibitor oligomycin, was shown to suppress The glycolytic inhibitor 2-DG, but not the OXPHOS inhibitor oligomycin, almost abolished this effect. In fact, metabolic reprogramming orchestrated by Notch1 through TAZ transcriptional activity may be not achieved only through transcriptional activation, but also through transcriptional repression of metabolic genes. Sardo et al. found that TAZ positively regulates glycolysis in NSCLC also through the transcriptional repression of PTEN [35].

Lactate produced by aerobic glycolysis has been implicated in the suppression of anticancer immune cells. Excessive amounts of lactate in the tumor microenvironment abrogate the release of lactate by glycolytic T cells by a gradient-dependent inhibitory mechanism. In addition, lactate uptake by macrophages results in the induction of Arginase-1 (ARG1) which in turn inhibits T cell activation [36, 37]. Here, we provided evidence to support a link between Notch1 signaling induced lactate and lung cancer immune escape through inhibition of cytotoxic T cell and NK cell activity. Our results show that Notch1 promotes aerobic glycolysis and facilitates immune escape through p300 or pCAF, which was enhanced by TAZ. It is expected to be a potential therapeutic target for an alternative treatment to enhance the outcome of immunotherapy in lung cancer. Thus, targeting Notch1 and TAZ may make lung cancer therapy more effective than targeting Notch1 or TAZ alone. However, further investigation of the optimal treatment regimen of double inhibition of Notch1 and TAZ is required.

## Conclusion

In conclusion, our study indicates that interaction between Notch1 and TAZ promotes aerobic glycolysis and immune escape in lung cancer. Targeting Notch1- TAZ axis could enhance the efficacy of immunotherapy through the inhibition of aerobic glycolysis in lung cancer.

## Supplementary information

supplementary table and figure legend

supplementary Fig 1

supplementary Fig 2

author-contribution-form

Reproducibility Checklist forms
