# Supplementary material for: Notch1/TAZ axis promotes aerobic glycolysis and immune escape in lung cancer
Source: Cell Death Dis. 2021 Sep 4;12(9):832. doi: 10.1038/s41419-021-04124-6 (PMC8418606; doi:10.1038/s41419-021-04124-6)
Supplement: Supplementary file 1 — supplementary table and figure legend [file 41419_2021_4124_MOESM1_ESM.docx]

**Supplementary table1-3 and supplementary figure legend**

**Table S1.** Peptides identified by mass spectrometry analysis of Notch1-containing protein complex that were eluted from A549 cells stably transfected with FLAG-tagged Notch1.

**TAZ**

K.SLLIATKISLQR.V

K.VGDGTYVVIAKR.I

K.ADTSSQALEDR.K

R.LSSGPSLSDSTS.D

K.VDVTYVIGGRSEK.D

K.RKTSSADAQLDSE.V

R.KADSQDATSSELER.V

R.LSSGSPSDPSERQTP.K

K.QFWHNMPRDEEIAST.I

K.VLLYIYSFGPADEEAVK.Q

K.IENLKIGDGRVTYVIGE.Q

K.GGNLSPQLSVRTPQTSP.K

R.SNCIGGAFQLIPENIYSK.V

K.VLNVLVTTTLYSALALPIATK.I

K.TESETPLNSTYSPGALDATGDFTVR.S

**p300**

K. TYEIYNPA.K

R.GWGVTQLIK.N

R.VYNTLGPAK.N

K.NLKEILNVVTYKT.V

R. YPTSESPSGLGLR.M

R. GRDLSSPVSPGSK.L

K.VLLYRNCVNILGGAK.R

K. TYKNVGGTGKLIGIGHR.I

R. TDMGLGATKTPFWAY.R

K. DGVQGNMSVFMEPS.S

K. GGLNNVPAKDSERSQ.V

K. SPPNDLENPPLGRYS.D

K. RTESSQDVTCNKYPIKAVN.E

K. DPTLVRGLPMYVEG.M

R. AKVIGKVVYDVSKAWL.K

R. AEIALKEGLTDTNQAG.S

**pCAF**

R. CEFCLKLYM.Y

R. PQYMSQPTPR.Q

R. SDRGQITEGLQSDF.E

R. NMSSETEQVVSGIK.T

K. VTPKLNYNVSCILTMNSF.K

K. QYNRVSN.A

R. DALDDPSSK.E

K. DSKSVQSIRPPSSQ.S

R. SATGKSLTNSPLDSGHS.D

K. AKLLQGNSPAEVN.I

K. AECKGNSPAEVLLQNAKIT.E

**Table S2.** Genes bound by TAZ and Notch1 in A549 cells from CHIP-Seq analysis.

| Btg2, Gli2, Gli3, Hes1, Hes5, Hey1, Id4, Jun, Nfatc4, Nr2e1 (Tlx), Pax6, Prdm16, Sall1, Sox2, Sox21, Sox9, Tcf3 (E2A/E47), Tcf7l2 (Tcf4), Tcfap2c, Tead2, Yap1, Zfhx4, Zhx2 |
| --- |
| Fzd2, Fzd8, Fzd9, Nrarp, Ppap2b, Sfrp1, Slc9a3r1 (NHERF1), Tcf7l2 (Tcf4) |
| Cdon, Gli2, Gli3, Smo |
| AI464131, Angptl2, Cntfr, Dusp16, E130112L23Rik, Epb4.1l5, Fgfr3, Grik2, Igfbp5, Jun, Ltbp3,Prdm16, Rcn3, Rlbp1, Spata13 |
| Casq1,Grik2, Ltbp3, Rcn3, Rhbdl3, Ttyh1 |
| Alk, Axl, Camk2d, D8Ertd82e, Ppap2b |
| Cdon, Celsr1, Epb4.1l5, Jub, Lamb2, Megf10, Tns3, Ttyh1 |
| Bcan, Igdcc4, Igfbp5, Kcnj10, Lamb2, Mfap2, Ptgfrn |
| Aldoc, Bcl2l11 (Bim), Ddit4 |
| Dbi, Aldoc, Ddit4, Prdm16, Slc27a1 |
| Hhipl1, HSPB6, Nfatc4 |
| Cbs, Chst3, Myo10, Pgpep1, Pgpep1, Ston2 |
| Bcl11b, Fezf2, Nr4a3, Sox5 |
| Abr, Adamts3, Agap2, Cck, Cnih2, Dab1 |
| Adamts3, Islr2, Tnr |
| Necab3 |
| Bcl11b, Cck |
| Accn1, Islr2, Slc17a7, Slit1 |

**Table S3.** Sequence information of the Hes1 promoter.

Hes1 promoter sequence for promoter activity assay
 TGGCTCCAGGAAAGTTTTTCAAAGTTCCCAGCAGCGTCTGCCCAGGTCGCCTCCGCGGGGCGAGCAGACGGCGGCAAGCGCGCCAGCCTCGCCGCCGCCTCTGCCGCCAGCAGAGCGCTCTGGGCGGCTCGCTCGCGGGAAGCGGGCCGAACTCCCGGCGGGCAGGCAGGCCCTCCTCCCGGGGCGAAAGCCG**CAGCTG**ACGCAGGCGGTTCGGAAGGCGGAAGCTGCCCCGCTCCGACCGCTCAGTC
AGCGCCGCGGCGCCTACACCTGGGGCCCCGACGCGCGGGCAAAGGCGCACGGCCCGGGGCGCCCGAGGGGGCGGTCCCCGCTGGGGGCCTCCAGGCGTCCCTGAGCAACGATCCCTTCCAAGTACCTCCCCGCACTCTCCCTTCCCTCCTGGCCCGAAGCTCCCGAGGGCGGGGGTTGGTGTGGGGCCCTGGTTCTTCTACGCCGCCCTGAGCATCCCGCTGCCCCCAACCCCTTCCAAGTTCCTCCTCGC
ACTACCCCCTCCCCAGCAACGTGAAGGGGAGGGGCGTGCCCAGGGTGAGCACGCCCTCTCATGAATATTAATAAGCGCGCATGCGCCCTGCCCGGCGTGCTGGGTAGAGGTGGCCAGCCCCGGCCGCTGCTGCCAGACGGGCTCTCCGGGTCCTTCTCCGAGAGCCGGGCGGGCACGCGTCATTGTGTTACCTGCGGCCGGCCCGCGAGCTAGGCTGGTTTTTTTTTTTCTCCCCTCCCTCCCCCCTTTTTCCATGCAGCTGATCTAAAAGGGAATAAAAGGCTGCGCATAATCATAATAATAAAAGAAGGGGAGCGCGAGAGAAGGAAAGAAAGCCGGGAGGTGGAAGAGGAGGGGGAGCGTCTCAAAGAAGCGATCAGAATAATAAAAGGAGGCCGGGCTCTTTGCCTTCTGGAACGGGCCGCTCTTGAA (-586~+345 from TSS)

**Figure S1. ChIP assay identified TAZ-binding sites in the Notch1 promoter.** Real-time PCR was performed to detect the amounts of immunoprecipitated products. The positive control: anti-RNA polymerase II; the negative control: normal mouse IgG. *: *P* < 0.05.

**Figure S2. Aerobic glycolysis is critical for Notch1/TAZ axis modulation of lung cancer cell growth.** (A) The proliferation curve of PC9 cells transfected with Notch1, TAZ, TAZ shRNA or empty vector. Cell proliferation was determined by the CCK-8 Kit. **P* < 0.05. (B) The proliferation curve of PC9 cells transfected with Notch1 or empty vector, treated with 2.5 mM 2-DG as indicated. Cell proliferation was determined by the CCK-8 Kit. **P* < 0.05. (C) The proliferation curve of PC9 cells transfected with TAZ or empty vector, treated with 2.5 mM 2-DG as indicated. Cell proliferation was determined by the CCK-8 Kit. **P* < 0.05. (D) The proliferation curve of PC9 cells transfected with Notch1, TAZ or empty vector, treated with 0.1 mM Oligomycin in normal culture medium (containing 25 mM glucose) as indicated. Cell proliferation was determined by the CCK-8 Kit. **P* < 0.05.
