# Supplementary material for: Notch1/TAZ axis promotes aerobic glycolysis and immune escape in lung cancer
Source: Cell Death Dis. 2021 Sep 4;12(9):832. doi: 10.1038/s41419-021-04124-6 (PMC8418606; doi:10.1038/s41419-021-04124-6)
Supplement: Supplementary file 4 — author-contribution-form [file 41419_2021_4124_MOESM4_ESM.pdf]

DECLARATION OF CONTRIBUTIONS TO ARTICLE

ADMC

Manuscript Number: CDDIS-20-4778R

Journal Name: *Cell Death & Disease* (the 'Journal')

Proposed Title of the Contribution:

Notch1/TAZ axis promotes aerobic glycolysis and immune escape in lung cancer (the 'Contribution)

Author(s):

Mian Xie, Xin-ge Fu, Ke Jiang (the 'Authors')

For all *CDDis* articles, each person named as an author in the published version must be able to show he or she has contributed substantially to the article.

Authorship credit should be based on 1) substantial contributions to conception and design, acquisition of data, or analysis and interpretation of data; 2) drafting the article or revising it critically for important intellectual content; and 3) final approval of the version to be published. Authors should meet conditions 1, 2 and 3.

Any person who cannot be shown to have made a substantial contribution to the article cannot be listed as an author in the final version. The name of any person who is deemed to have made a minor contribution can, however, appear in the Acknowledgments section of the article.

Please complete the table below to indicate the contributions of all named authors to the manuscript.

Author Full Name:	Specification of Contribution to the Manuscript:
Mian Xie	Conception and design, Development of methodology, Analysis and interpretation of data, Writing, review, and/or revision of the manuscript:
Xin-ge Fu	Conception and design, Development of methodology
Ke Jiang	Acquisition of data

Please complete the table below to indicate the contributions of all named authors to the figures.

Figure 1:

Mian Xie, Xin-ge Fu

Figure 2:

Mian Xie, Xin-ge Fu

Figure 3:

Mian Xie, Xin-ge Fu

Figure 4:

Mian Xie, Xin-ge Fu

Figure 5:

Mian Xie, Xin-ge Fu

Figure 6:

Mian Xie, Xin-ge Fu

Signed for and on behalf of the Author(s):	Print Name:	Date:
	Mian Xie	May 27, 2021
